# Excipients in the Paediatric Population: A Review

**DOI:** 10.3390/pharmaceutics13030387

**Published:** 2021-03-13

**Authors:** Khadija Rouaz, Blanca Chiclana-Rodríguez, Anna Nardi-Ricart, Marc Suñé-Pou, Dèbora Mercadé-Frutos, Josep María Suñé-Negre, Pilar Pérez-Lozano, Encarna García-Montoya

**Affiliations:** 1 Department of Pharmacy and Pharmaceutical Technology and Physical Chemistry, Faculty of Pharmacy and Food Sciences, University of Barcelona, Av. Joan XXIII, 27-31, 08028 Barcelona, Spain; khadirouaz@gmail.com (K.R.); annanardi@ub.edu (A.N.-R.); msunepou@gmail.com (M.S.-P.); debora.mercade@ub.edu (D.M.-F.); jmsune@ub.edu (J.M.S.-N.); perezlo@ub.edu (P.P.-L.); encarnagarcia@ub.edu (E.G.-M.); 2 Pharmacotherapy, Pharmacogenetics and Pharmaceutical Technology Research Group Bellvitge Biomedical Research Institute (IDIBELL), Av. Gran via de l’Hospitalet, 199-203, 08090 Barcelona, Spain

**Keywords:** excipients, paediatrics, security, toxicology, STEP and ODT

## Abstract

This theoretical study seeks to critically review the use of excipients in the paediatric population. This study is based on the rules and recommendations of European and American drug regulatory agencies. On the one hand, this review describes the most frequent excipients used in paediatric medicine formulations, identifying the compounds that scientific literature has marked as potentially harmful regarding the side effects generated after exposure. On the other hand, this review also highlights the importance of carrying out safety -checks on the excipients, which, in most cases, are linked to toxicity studies. An excipient in the compilation of paediatric population databases is expected to target safety and toxicity, as in the STEP database. Finally, a promising pharmaceutical form for child population, ODT (Orally Disintegrating Tablets), will be studied.

## 1. Introduction

The scientific literature suggests that most commercialized drugs are not suitable to be used on the paediatric population, as they are presented in an inappropriate pharmaceutical dosage or form, or because of the excipients they contain. In the face of this reality, compounding is the alternative for paediatric patients. Auxiliary substances or excipients should be used in the development of a compounding formula in order to allow the drug to be administered in an easily and personalized manner. By doing so, the active ingredient will be formulated in a stable, effective, and safe form [1].

The process of formulating excipients in paediatrics is a complicated task that requires various considerations to be accounted for in order to for them to be appropriate; variables such as an acceptable taste, age, dosage forms, among others, must be taken into account when selecting safe excipients. Furthermore, children’s rapid growth and development are associated with changes in various organs, body composition, protein bonds, active transport mechanisms and metabolic pathways, which must also be taken into account [2]. In addition to being a complicated task, it is also a critical step in the development of paediatric formulations, as some acceptable excipients in formulations for adult patients are not suitable for paediatric use.

It is thus of particular relevance to carry out an assessment of the safety of excipients prior to their use in paediatrics. Indeed, Georg Schmitt [3] advocates for non-clinical safety studies being carried out in juvenile animals to assess excipient toxicity or sensibility and also to establish safe exposures in paediatric age groups. He specifically recommends that excipient toxicity studies also be carried out, as they provide a detailed assessment of clinical risk. He further suggests that even excipients with significant toxic potential for children may be acceptable after a rigorous assessment of the risk they pose is made. Another factor to be considered for toxicological studies is the extent to which the target disease may be alleviated by the formulation of that medicine. Thus, pharmaceutical companies should filter the demands for safety assessments by selecting those that will contribute to a potential therapeutic benefit, while helping to develop a reference list of excipients generally considered safe for use in paediatric formulations. In this way, the clinical decision-making process will be made easier.

This theoretical study’s main objective is to critically review the use of excipients in paediatrics with an emphasis on the issue of safety, mainly on the basis of toxicological studies. This will enable information to be obtained that will allow decisions to be made regarding the masterful preparation of formulations. This study also seeks to investigate the development of databases and initiatives in order to record corroborated information on excipients for paediatric use, thus serving as a guide for clinical professionals. 

To do this, databases such as Web of Science, PubMed, SciFinder and SciFindern Search, as well as books related to the subject, were consulted. Please note that most of the selected literature is from the last two decades. Subsequently, six tables were created to provide details on the data obtained:

- Table 1. Toxicity database.
- Table A1. Most important characteristics of the excipients discussed in this review (in alphabetical order).
- Table A2. Examples of solid and semi-solid medicines used in Spain for the paediatric population: List of excipients and relevant characteristics of the pharmaceutical form (PF) (performed consultation of CIMA database, September 2020).
- Table A3. Examples of liquid medicines used in paediatrics: List of excipients and relevant characteristics of (PF).
- Table A4. Examples of FDA-registered drugs used in paediatrics (FDA database and DAILYMED October 2020).
- Table A5. Examples of liquid formulations for paediatric use in research articles.

## 2. Paediatric Regulatory Context

Changes in physical, metabolic and psychological processes that occur during children’s growth, from birth to adulthood, suggest that children should not be considered as young adults, and nor should they be grouped as a single group. Rather, the pharmaceutical development of paediatric drugs should focus on several acceptable dosage forms that are able to meet the needs of most children in different age groups. This can be achieved by developing dosage forms which facilitate the administration of a dose range which would vary according to the child’s age and/or other important parameters [4]. 

Before there were regulations for the development of paediatric drugs, children were known as “therapeutic orphans”. They lost the advances of conventional medicine, since the vast majority of advances were aimed at the adult population, and there were not many approved medicines for children. Children were treated with approved drugs following successful studies on adults, but with few or no trials on the paediatric population (off-label use). The large number of subsequent issues with clinical trials on children, as well as the need for drug authorization in the paediatric population, among other reasons, were the driving factors for the creation of a legislative and regulatory framework for clinical studies in paediatrics. The US pioneered these in the late 1980s, and with the adoption of these paediatric regulatory initiatives, significant improvements were made [4].

It was only in 1997 that European regulators agreed to strengthen legislation on the use of new medicines in children. In 2000, European health ministers asked the European Commission to make proposals for a legislation to ensure that new paediatric medicines placed on the market were tailored to the specific needs of children. In 2004, after a major debate, a regulatory bill was issued, which took into account lessons learned from paediatric regulation that the US was already addressing [5]. On 26 January 2007, the Paediatric Regulation entered into force in the European Union, and focused mainly on regulating the development of paediatric formulations for children between 0 and 18 years of age, but also sought to:

- Ensure that these medicines were of good quality.
- Verify that paediatric medicines were produced following ethical and legitimate research, that children were not subjected to unnecessary trials.
- Improve the accessibility and availability of information on drug use in the paediatric population.
- Such regulations led to the establishment of the Paediatric Committee (PDCO), whose main function was to regulate the studies that companies should conduct in children as part of a Paediatric Research Plan (PRP) [6].
- The Paediatric Regulation consists of [7]:
- Regulation (EC) 1901/2006 of the European Parliament and of the Council on medicinal products for paediatric use; and
- Regulation (EC) 1902/2006, an amending regulation in which changes were made to the original text in relation to the European Commission’s decision-making procedures.

In October 2017, the European Commission published a ten-year report on the implementation of the Paediatric Regulation. The report showed an increase in medicines for children in most therapeutic areas over the past ten years, especially in rheumatology and infectious diseases. However, in rare diseases, progression was lower. A report on the first five years was also published in June 2013, which concluded that paediatric development had become a more integral part of the overall development of medicines in the European Union [4,8].

The European Guideline on pharmaceutical development of medicines for paediatric used [4] offers several tips for paediatric drug formulation.

Excipients in a paediatric formulation should be chosen appropriately, avoiding any excipients that are potentially toxic or unsuitable for children. Choosing the right excipients in the development of a new paediatric drug is one of the most important aspects, as it requires special safety considerations. In general, the following aspects should be taken into account when selecting an appropriate excipient for a paediatric medicinal product [4]:

- Excipient function in formulation and possible alternatives.
- Safety profile of the excipient for children in target age groups, based on a unique and daily exposure.
- Expected duration of treatment: short term (a single dose for a few days) or long term (weeks and/or months).
- Severity of the condition to be treated and therapeutic alternatives.
- Patient acceptability, including palatability.
- Allergies and sensitization. Children suffer from sensitization problems more commonly than adults. Applicants should avoid, when possible, excipients with known potential to cause sensitization or allergies.

If the use of any excipient in the formulation that produces or may pose any risk to the child cannot be avoided, the added value of the chosen pharmaceutical form of dosing (and the route of administration) should be balanced with the possible use of another. However, security issues can only become apparent when the product is used on a larger scale.

Furthermore, the first joint paediatric regulatory action was taken by the ICH (The International Council for Harmonization of Technical Requirements for Pharmaceuticals for Human Use), an organization working on harmonizing drug regulation requirements between the EU, Japan and the US. In July 2000 Guideline E11 (R1) was published: Clinical investigation of medicinal products in the paediatric population, with the final version in August 2017 [9].

The objectives of this guide were to encourage and facilitate the development of paediatric medicines at the international level, as well as to provide a summary of critical problems in the development of these medicines and new approaches to their safe, efficient and ethical clinical study. ICH E11 became an important tool in the design of paediatric clinical research worldwide, providing guidelines (rather than proscribing practice) [9,10].

The WHO launched the initiative Making Medicines Child Size in 2008 to issue a list of essential medicines for children, betting on quality paediatric development and adequate access of these medicines to the entire paediatric population, in particular underdeveloped countries [11]. The most current one is the 7th edition, which was published in 2019 (WHO model list of essential medicines for children) [12].

In the early 1980s, the FDA (Food and Drug Administration) began taking steps to provide incentives to the pharmaceutical industry for the development of paediatric drugs. In 1994, the Paediatric Labelling Rule was issued, requiring the authorization of a new paediatric drug to be supported by safety and efficacy data to support its use. However, that rule was not mandatory and was unsuccessful. For this reason, the US-FDA proposed in 1998 Paediatric rule which proposed to guarantee the above-mentioned objectives, both at and after the approval of the new drug [13].

It should also be noted that the FDA (Nonclinical Studies for the Safety Evaluation of Pharmaceutical Excipients) published a document that provides guidance on the development of safety profiles to support the use of new excipients as components of drugs or biological products, which could be applied in paediatric experiments [14,15].

### Examples of Databases and Initiatives for the Registration of Information on Excipients Used in the Paediatric Population

It is certainly necessary to take into account the safety of excipients used in paediatric products, as the toxicity of these excipients may differ from that of adults [16]. Under this assumption, it is essential to develop methodologies that provide an integrated assessment of exposure to potentially toxic excipients contained in medicines. Therefore, in 2007, members of pharmaceutical industries, hospitals and academics interested in improving drug formulations in paediatrics founded the European Paediatric Formulation Initiative (EUPFI). The latter sought to address safety problems linked to excipients used in children [17], as well as the development of platforms for the systematic evaluation of excipients in new-borns [18].

EuPFI is currently a consortium of 10 pharmaceutical companies, 5 universities, 1 hospital and, exclusively, the European Medicines Agency (EMA) as an observer. The goals and objectives of this consortium are summarized in [19]:

1. Identify the problems and challenges associated with the development of paediatric formulation and consider ways to obtain better medicines and dosage forms clinically relevant to children.
2. Promote early pharmaceutical consideration for the development of paediatric medicines.
3. Identify potential information and knowledge gaps in the development of paediatric formulations.
4. Improve the availability of information from paediatric formulations.

The scientific literature shows that excipients commonly used in adult medicines have been associated with high toxicological risks and safety problems in children [20]. Following the United States Paediatric Formulation Initiative (USPFI) and Global Paediatric Research (GRIP), the Paediatric Excipient Safety and Toxicity Database (STEP) was created to address the need for effortless access to information about the excipients’ safety and toxicity [21]. The STEP database is presented as a resource of information to facilitate access to data on the use and acceptability of excipients in children, thus allowing a rapid evaluation of the risks due to the use of certain excipients in the paediatric population and an improvement in the scientific decision making [2,22]. Furthermore, the STEP database provides comprehensive and comparative information on the safe use and acceptability of excipients in paediatrics. For the reasons listed above, the STEP database stands out with respect to other existing public resources (such as TOXNET) or databases (such as Vitic or ACToR) that organize their informational content in free text format, thus preventing data from being filtered as needed (see Table 1) [23].

In general, the above purposes go in line with increasing the number of excipients registered in the database to be useful in practical research. Therefore, the following selection criteria were considered for excipients of interest [2]:

1. Excipients known to be toxic/have general safety issues.
2. Frequency of appearance as contaminants or toxics in paediatrics (where applicable).
3. Evidence in the toxicity literature in paediatrics. The above criteria were applied to identify excipients for inclusion in the STEP database. Excipients were shortlisted/prioritized through surveys within EU and US PFI members.

According to the above criteria, in the development of databases on the safety and toxicity of excipients in the paediatric population, the following are prioritized, as they are most likely to cause damage and side effects in this population [2]:

1. Propylene glycol (PG)
2. Ethanol
3. Polysorbate 80
4. Benzyl alcohol
5. Parabens (propyl, methyl, ethyl and butyl)
6. Benzalkonium chloride
7. Aspartame
8. Sorbitol
9. Benzoic acid
10. Sodium benzoate

In 2014, the first version of the STEP database was launched for the systematic evaluation of its integrity, quality, configurability, usability, and maintainability under the daily practices of the different and diverse professionals who use it. After launch, a validation study of the tool was initiated with the following objectives [2]:

1. Validate the STEP Version 1 database against the potential needs of end users to ensure that the STEP database meets users’ expectations.
2. Evaluate the functionality and usability of data application by
  - Ensuring proper ease of use (navigation), understanding and user satisfaction.
  - Characterizing how easy it is to perform a task using the database.
  - Identifying problems in interaction with systems.
3. Evaluate the impact of this database on the development of paediatric medicines.
4. Establish viable recommendations to further improve the functionality of the system and increase its beneficial effects on the development of paediatric medicines.

The results of the validation study identified different database usage issues, which are grouped into three areas: I. Content and presentation of results; II. Adequacy of the database to the characteristics of different users, navigation features; and III. Search. Many of the problems observed might have happened due to assuming that users would have sufficient knowledge, therefore some elements were not clearly exposed for the new user to understand. Furthermore, users with limited computer skills may also find the registration process confusing. These issues involved changes and improvements to STEP design and functionality, making it a more efficient database when deriving from a Version 2 [21].

To perform an adequate risk/benefit assessment of the current medication standard, it is necessary to compare the daily amount of excipients in the most vulnerable patient with clinically established safety levels for the same age group. The SEEN project is an example of this, as it developed a retrospective cohort study, with neonatal patients (age 5 or younger) treated with multiple medicines. Preparations were recorded with ethanol, propylene glycol, benzyl alcohol, parabens, aspartame, glycerol, sorbitol and polysorbate-80 and cumulative amounts [24] were calculated.

The results obtained demonstrated limited knowledge about the acceptability of different dosage forms, flavours and, more importantly, the safety of formulation excipients in relation to the age and stage of development of children [24].

## 3. Excipients: Functions and Main Adverse Effects 

Paediatric formulations need excipients to maintain their quality and promote the acceptability of childhood patients [25]. However, just because they are necessary does not mean that they are toxicity-free products; in fact, a study by Georgi and collaborators [26,27] confirms that many of the medicines used in paediatrics contain some toxic or potentially toxic excipient for the paediatric population, with this data being present in two-thirds of new-borns in 21 European countries. Thus, excipients used in paediatric formulations require a thorough assessment of short-term and long-term safety prior to their use in these formulations [28]. A classification of the main excipients will then be developed according to the role they play in the formulation, mentioning the possible adverse effects on the paediatric population. Furthermore, a summary appendix (Appendix B (Table A1)) of the excipients discussed in this paper will be prepared.

### 3.1. Diluents 

Lactose, starch and microcrystalline cellulose are often used as diluents, as they are generally safe in the adult population. 

#### 3.1.1. Lactose 

Lactose, which is a mandatory excipient, is recommended not to be used in patients with lactose intolerance and is contraindicated in patients with galactosemia [1]. It may cause hypersensitivity reactions in children and new-borns. Infants with lactose intolerance do not properly metabolize lactose, due to the deficiency of the enzyme lactase, thus causing the accumulation of lactic acid, hydrogen and carbon dioxide. Symptoms such as severe abdominal pain, flatulence, bloating or swelling and diarrhoea may, therefore, appear, as well as systemic symptoms such as muscle, joint pain and eczema [28]. It should be noted that children may sometimes have very severe and prolonged reactions to lactose that can lead to additional complications, such as dehydration, bacterial proliferation and metabolic acidosis [1,28].

Starch, dehydrated calcium hydrogen phosphate, erythritol and cellulose powder are alternatives to lactose in paediatric formulations. They have lactose-like flow properties and produce tablets that can disaggregate in a time less than lactose [28].

#### 3.1.2. Starch

Starch is one of the most commonly used excipients and, in addition to being a diluent, it has binder and disintegrating properties. Due to its properties, starch should be preserved in a dry environment, as it can be an excellent growing medium for microorganisms in case of moisture, which may cause microbiological contaminations. In addition, it may give proliferation of carcinogenic aflatoxins, if contaminated by two species of fungi closely enhanced by each other: Aspergillus flavus and Aspergillus parasiticus [29]. 

#### 3.1.3. Microcrystalline Cellulose

Microcrystalline cellulose is a partially depolymerized purified cellulose that is presented as a white, odourless and tasteless crystalline powder composed of porous particles. It is commercially available in different particle sizes and moisture grades that have different properties and applications. It is considered a relatively non-toxic and non-irritating material. It is not absorbed systemically after oral administration and therefore has little toxic potential [29,30].

Microcrystalline cellulose is used in pharmaceutical products, mainly as a binder and thinner in tablet and oral capsule formulations. In addition to its use as a binder and thinner, it also has some lubricating and disintegrating properties that make it useful for forming tablets [30].

### 3.2. Solvents 

Some of the most common solvents are water, ethyl alcohol, propylene glycol (PG), glycerol and polyethylene glycol [28,29].

#### 3.2.1. Water

Water is the most commonly used agent in paediatric formulations, as liquid preparations are easier to administrate and allow a more accurate dose adjustment [1,29]. Water is an ideal medium for the proliferation of microorganisms (bacteria and fungi) despite their purification, which is why antimicrobial agents have to be added.

In paediatric oral formulations, the total volume of fluid is of vital importance for the taste and ability to adequately measure the volume to be administered: in children under 5 years of age a volume of less than 5 mL should be administered and, in children under 10 years of age, a volume of less than 10 mL [29] should be administered.

#### 3.2.2. Ethyl Alcohol (Ethanol)

Ethanol is one of the excipients of concern to international health regulatory agencies, as it causes neurotoxicity and cardiovascular problems in the paediatric population; it is a potentially harmful excipient in neonates. For this reason, permissible maximum limits have been set and, in some countries, non-alcoholic medicines are to be established. It is a very permeable excipient with regard to the blood–brain barrier, and the one most commonly used in oral medicinal products, reaching 63% of cases [26]. It is rapidly absorbed into the gastrointestinal tract and is primarily metabolized in the liver to acetaldehyde, which is oxidized to acetate [29].

Indeed, Macrel and Bernando’s review of liquid formulations in Brazil has furthered our understanding of the high use of ethanol. These researchers demonstrated that ethanol is used in various concentrations and functions: as solvent (main function), co-saver, flavouring agent, preservative and as an extraction solvent in herbal medicines [26,27]. It also has antimicrobial properties and increases the permeability of many preparations [29].

The use of ethanol as an excipient carries potential hazards and adverse effects, which are already observed at a dose of 100 mg/dL. These effects include hypoglycaemia, acidosis and hydro-electrolytic alterations. Very high intake can lead to stupor, coma, respiratory depression and cardiovascular collapse. Hypoglycaemic seizures may also occur in children [29,31]. For all these side effects, any alcohol should be avoided in paediatric forms. However, it is still used in many liquid preparations, because it is the only solvent that allows the solubilization of certain active substances [29].

In both the United States and the European Union, guidance on maximum ethanol limits in medicinal formulations is increasing [17]. According to the World Health Organization and a regulation existing in the United States, the maximum alcohol content in paediatric formulations should not exceed the limits specified in Table A1 [29,31,32].

It should be noted that ethanol was also able to interact with many active substances of other medicines that the child is taking [29] and, therefore, possible interactions must be studied prior to concomitant administration. Furthermore, new contributions in the scientific literature on excipients, including ethanol, is expected to help health professionals predict the risks of using a particular excipient, especially in the paediatric population. For example, the guideline excipients in the label and package leaflet of medicinal products for human use alerts on the risk of the use of ethanol and proposes changes on its use.

#### 3.2.3. Propylene Glycol (PG)

PG is used as a solvent to stabilize substances that are not water soluble, in parenteral and non-parenteral formulations. It also has moisturizing, antimicrobial properties and can be used as plasticizer. It is rapidly absorbed through the gastrointestinal tract and damaged skin and metabolized in the liver to lactic acid and pyruvic acid [29].

Exposure to high doses of PG may affect the Central Nervous System, especially in new-borns and children under 4 years of age [29]. Due to children’s physiological and metabolic immaturity, PG can accumulate rapidly causing toxicity [33]. In new-borns, its half-life is very long, almost seventeen hours, compared to that of adults, which is about five hours [29]. The GRAS (Generally Recognized as Safe) classification of excipients typically does not consider the differences in physiological and metabolic maturation between the paediatric and adult populations [33], a fact that justifies some important adverse reactions presented by PG in the paediatric population [29]:

- Hyperosmolar syndrome in burnt children with topical arsenic sulfadiazine ointment containing PG.
- Precipitation of irreversible deafness in pretermits who received a multivitamin complex containing PG.
- Parenterally it is possible to observe haemolysis, seizures, respiratory depression, hypertension.
- Contact dermatitis is topically observed.

In the 1980s, cases of biochemical abnormalities, including hyperosmolarity, lactic acidosis and elevated levels of creatinine and bilirubin, were documented after exposure to 3 g/day of PG and for at least 5 consecutive days. Clinical symptoms, including seizures and bradycardia episodes [33], then appeared. In 2011, the U.S. FDA reported health problems in premature new-borns associated with the use of Kaletra^®^ (lopinavir/ritonavir) solution; liquid preparation containing high amounts of PG and ethanol [33,34].

Exposure to PG in new-borns and children under 4 years of age remains common, despite historical and contemporary reports dealing with toxic adverse effects of this excipient. Thus, the study of Allegaert J. [33] in terms of the PG research project in new-borns is of great interest, as it provides scientific evidence on the tolerance and plasma clearance of this excipient, including differences in elimination pathways (renal pathway compared to the hepatic pathway).

#### 3.2.4. Glycerol 

Glycerol, a mandatory excipient (E-422), is used as solvent, sweetener, viscosizer and preservative.

When used at high concentrations (more than 40%), it can cause mucositis in the stomach, as well as diarrhoea and electrolyte disturbances due to its hygroscopic and osmotic properties. Therefore, a maximum amount of 10 g/dose [1,29] has been established.

In the adult population glycerol has few adverse effects. However, cases of neurological toxicity have been reported in the paediatric population [29].

#### 3.2.5. Polyethylene Glycol (PEG)

PEG is a polar and water-soluble substance used as a co-solvent, suspensor and viscosity agent. The PEG 400 is the most used in liquid formulations. It may cause some laxative effect when taken orally, with the maximum daily dose established in adults at 10 mg/kg/day [1]. 

PEG has low oral bioavailability and renal elimination. Due to its properties, significant adverse effects such as diarrhoea and nephrotoxicity have been reported, so the maximum recommended daily dose is 10 mg/kg body weight [1]. It can also cause some laxative effect when taken orally. When new-borns and infants are exposed to high doses of PEG, gastrointestinal disorders, adverse effects typical of alcoholic solvents may occur [1,28]. 

### 3.3. Coating Agents

#### Phthalates

Phthalates play a primary role as a coating agent (film-forming, plasticizer) in medicinal formulations.

Exposure of pregnant women to phthalates has been associated with abnormalities in the development of the foetus, such as cleft palate and skeletal malformations; abnormalities that can end in stillbirth. It was observed that they have a high potential to produce toxicity in the development of experimental animals, as well as in their reproduction [28].

Due to these risks of certain phthalates to health, in March 2012, the CDER published a guide to orient the pharmaceutical industry on the use of phthalates: “Limiting the use of certain phthalates as excipients in CDER regulated products”. This guidance document recommends limiting the use of certain phthalates, such as dibutyl phthalate (DBP) and di(2-ethylhexyl) phthalate (DEHP) [28].

### 3.4. Preservatives

Preservatives are a group of excipients that prevent microbial growth and, consequently, the degradation of the active substance and the possible alteration of the organoleptic characteristics of the final formula [35].

The American Academy of Paediatrics does not recommend the use of preservatives in reparations for patients under 3 years of age due to the lack of physiological and metabolic maturation of these patients. This lack of maturation may lead to the accumulation of preservatives in the liver, a fact that increases the risk of cardiovascular collapse, in addition to producing non-specific reactions or even allergies [1,35]. It should be noted that preservatives are not contraindicated in children under 3 years of age, but should only be used in imperative cases [1].

#### 3.4.1. Sodium Benzoate 

Sodium benzoate is a preservative widely used in pharmaceutical and cosmetic formulations, at concentrations between 0.02% and 0.05% [29]. Its maximum activity occurs in weakly acidic pH 4.5 solutions and is inactive at pH values greater than 5 [35].

As side effects, it can cause contact hives and other allergies. In premature children, its use is contraindicated, as it presents a risk of metabolic acidosis and jaundice [29,35].

One of the large prospective studies conducted by Nellis and collaborators [36,37] in hospitalized neonates in Europe described the administration of eight potentially harmful excipients of interest (EOI) (parabens, polysorbate 80, propylene glycol, benzoates, sodium saccharine, sorbitol, ethanol and benzalkonium chloride) and identified risk factors resulting from exposure. Neonates appear to lack the ability to conjugate benzoates with glycine, leading to the accumulation of benzoic acid that can cause metabolic acidosis and neurotoxicity [26,27].

The ESNEE (European Study of Neonatal Exposure to Excipients) clinical study [38] showed that sodium benzoate was found in 10 medicines given to new-borns, despite being a highly toxic excipient to them. Preservatives such as parabens (and their sodium salts) and propyl para-hydroxy-benzoate were also found in 24 paediatric medications, and ethanol in 8.

#### 3.4.2. Benzyl Alcohol 

Benzyl alcohol presents antibacterial properties. For that reason, it is used as a preservative in a lot of medicines. Its activity depends on the pH; being at it is maximum at a low pH (between 2.5–4.5). It is used at the concentration of 0.01–0.15% in oral preparations [35].

In adults, it is metabolized to benzoic acid, which is conjugated in the liver with glycine. As a result, the acid hippuric formed is excreted in urine. However, in new-borns, this conversion of the benzoic acid into hippuric acid is very diminished, because of the lack of liver maturation. That justifies fatal intoxication cases in new-borns who had their umbilical catheters cleaned with benzoic acid. Consequently, cases of metabolic acidosis and respiratory depression occurred. Additionally, other adverse effects have been described, like intraventricular bleeding, cerebral palsy and developmental delay. In some cases, there have been reactions of hypersensitivity, allergy and contact dermatitis [29,39,40,41].

In the 1990s, Svinning and collaborators [42] conducted a review of the medical records of babies who weighed less than 1250 g at birth and were admitted to the neonatal intensive care unit. The main objective of this study was to assess the impact of the toxicity of benzyl alcohol, following discontinuation of the use of solutions to wash intravascular catheters containing benzyl alcohol. A significant decrease in mortality rate and incidence of Grade III/IV intraventricular haemorrhage was observed among infants weighing less than 1000 g at birth who were not exposed to benzyl alcohol (as opposed to those who were). 

The maximum dose of benzoic acid (and other benzoates, calculated as benzoic acid) recommended by WHO is 5 mg/kg body weight per day in adults, a dose that, in children, logically, should be much lower [29,35]. As the effects on new-borns are severely toxic, the U.S. FDA has recommended the exclusion of benzyl alcohol from medications, intravenous fluids, and heparin washing solutions for them [36]. The EMA states that any medicine containing benzyl alcohol “should not be given to premature babies and new-borns” [42,43]. In fact, currently, any exposure to benzyl alcohol is contraindicated in children under 3 years of age [44].

#### 3.4.3. Benzalkonium Chloride

Benzalkonium chloride is a quaternary ammonium used in ophthalmic preparations at a concentration of 0.01–0.02% (*v/v*). Generally, it is non-irritating or sensitizing and is well tolerated in skin solutions.

As a side effect, it can cause bronchoconstriction in asthmatic patients, if used in nebulization solutions. Furthermore, cases of ototoxicity may occur in otic preparations, hypersensitivity in topical skin preparations and respiratory failure in infants who ingest this excipient, with this side effect being the most severe [29].

#### 3.4.4. Thiomersal

Thiomersal is a preservative widely used in vaccines and topical preparations, such as eye drops. Its toxicity is similar to mercury: in fact, it contains a mercury atom in its molecular structure. The concentration used depends on the medicinal product: in injectable preparations 0.01% is used and in ophthalmic solutions between 0.001% and 0.15% [30]. 

Several allergic hypersensitivity reactions (e.g., erythema, vesicles) have been reported. Therefore, health authorities have recommended their withdrawal from vaccines at risk of toxicity. Recently, thiomersal has also been implicated in the onset of autism spectrum disorders in children who received aluminium salt vaccines as an adjuvant. Accordingly, various countries (including Spain) no longer market paediatric vaccines with this component [29]. The use of single-dose vials is recommended in many cases to prevent the use of preservatives such as thiomersal or sulphites such as sodium metabisulphite [28].

#### 3.4.5. Parabens

Parabens are the most commonly used preservatives (also in cosmetics and foods), due to their wide antimicrobial spectrum and their effectiveness over a very wide pH range (between 4 and 8) [29,35]. 

Parabens are of mandatory declaration. They are used at concentrations between 0.01 and 0.2% [45], although it is most common to use a mixture in proportion 10:1 (0.2% methylparaben + 0.02% propylparaben). The maximum recommended daily dose is 10 mg/kg body weight [35].

They may produce a cross-hypersensitivity reaction in patients allergic to aspirin. This is because the main metabolite of parabens is hydroxyparabenzoic acid, structurally very similar to aspirin [29].

Recent pharmacovigilance studies have highlighted certain questions about the purported safety (non-teratogenic or carcinogenic) of parabens [29]. Alternatives should therefore be found, especially in paediatric formulations. Antimicrobials are not necessary for parenteral formulations. The absence of parabens and benzoates in 85% of parenteral prescriptions suggests that administration of these excipients can be largely avoided [36]. 

### 3.5. Antioxidants 

Antioxidants are a group of chemical compounds used to prevent oxidation of the active substances in formulations [29].

#### 3.5.1. Sulphites

Sulphites are antioxidants widely used in different formulations; sodium sulphite, sodium bisulfite, sodium metabisulphite and potassium metasulfite [29] are the most common.

Regulatory agencies (e.g., FDA, EMA) consider excipient sulphites safe. However, they present risks and possible fatal side effects derived of their use. One of the most common cases occurs in asthmatic patients, who may develop severe bronchospasm if they take medicines containing sulphites in their formulation [29].

The antioxidants constitute a group of compound chemists used to avoid the oxidation of the active principles in the formulations [29].

It should be noted that a large number of people are sensitive to sulphites and may experience a variety of symptoms, including dermatological, gastrointestinal and respiratory symptoms. However, reactions that develop in the respiratory tract explain most cases of sensitivity to sulphites. It is important to note that several individuals experience a variety of symptoms after exposure to sulphites; therefore, skin, intestinal and respiratory reactions can occur simultaneously and in various combinations and severity. People with sensitive skin who regularly use cosmetics or topical medications containing sulphites have chronic skin symptoms, especially on the hands, perineum and face. Sensitivity to sulphites is a very real problem that significantly affects the health of many people, especially asthmatics. Sensitivity to sulphites should be considered when people show adverse reactions to a variety of exposures, without an obvious pattern, particularly when those people experience worsening asthma symptoms after consumption of foods such as dried fruits and wines, or adverse skin reactions, after the use of cosmetics or medicinal creams [46].

#### 3.5.2. Propyl Gallate

Propyl gallate is an antioxidant used to prevent the breakdown of fatty acids. It is used at a concentration of 0.1% and also has a synergistic effect with other antioxidants. In neonates it can cause dermatitis, skin allergy and methemoglobinemia [29].

### 3.6. Sweeteners 

The use of sweeteners varies between routes of administration and, like preservatives, are not necessary in parenteral administrations [36,37]. They have been linked to photosensitivity reactions, diarrhoea and poor absorption of nutrients [36,47].

The most commonly used sweeteners in pharmaceutical formulations are sucrose, sorbitol, mannitol, aspartame and sucralose.

#### 3.6.1. Sucrose 

Sucrose is a natural disaccharide that is hydrolysed in the gut into two monosaccharides: glucose and fructose.

In children with type I diabetes, the use of sucrose should be avoided. Very high concentrations (up to 35% are used for liquid formulations such as syrups). When the patient needs prolonged treatment with these preparations, he or she is at risk of dental damage. It has also been described that administration at very high doses on a daily basis may be carcinogenic [29].

#### 3.6.2. Sorbitol 

Sorbitol is a monosaccharide that is not absorbed into the digestive tract and is therefore considered safe in paediatric patients, although it is laxative at high doses. It is also used as a diluent as well as capsule plasticizer [29].

Sorbitol is another example of an excipient that causes gastrointestinal disorders, such as abdominal pain, swelling, flatulence, vomiting and osmotic diarrhoea. Because sorbitol is metabolized to fructose, it should be avoided on children with fructose intolerance and hypoglycaemia. In isolated cases it can cause liver damage leading to coma and even death [28,29,30].

In infants the accumulation of sorbitol can lead to diabetic complications such as retinopathy and cataracts. Therefore, the amount of sorbitol is limited to 0.3 mg/kg in paediatric formulations [28].

#### 3.6.3. Mannitol

Mannitol is used as a sweetener and as a diluent. It has been linked to severe anaphylactic reactions in paediatrics [29]. As in the case of sorbitol, it is not absorbed into the digestive tract, so it has laxative properties at high doses.

#### 3.6.4. Aspartame

Aspartame is an artificial sweetener that has 180 and 200 times more sweetener power than sucrose. Because of this, it is the most used sweetener in the pharmaceutical and food industry. It is a disaccharide made of an aspartic acid and a methyl phenylalanine ester. It is an excipient of mandatory declaration and its maximum dose has been set at 40 mg/kg body weight [29,35].

Phenylalanine is very harmful for patients with phenylketonuria, as well as for pregnant mothers who carry a foetus of such metabolopathy. The use of aspartame in patients with phenylketonuria should be avoided. The adverse effects of aspartame that have been described are: neurological (neurotoxicity, epilepsy, headache, panic attack and hallucinations), hypersensitivity reactions (vascular and granulomatous panniculitis) and cross-reaction with sulphonamides [29]. 

#### 3.6.5. Saccharine

Saccharine is also an artificial sweetener 300–600 times stronger that sucrose, but if not used properly it can leave a residual bitter taste. Your daily dose should not exceed 2.5 mg/kg body weight. It is recommended to limit the daily dose in children and pregnant women [29,48].

Currently, controversy about its safety remains present, as in adults it has been linked to bladder cancer when used at very high doses. Adverse effects of saccharine include hives, itching, photosensibilization, eczema, as well as nausea and diarrhoea [29].

#### 3.6.6. Sucralose

Sucralose has a sweetener power between 100 and 300 times higher than sucrose. Its maximum daily dose is 15 mg/kg in weight.

Sucralose is a non-toxic compound and is also not irritating, but it is not considered totally inert. It can increase the expression of cell flow transport protein glycoprotein P and two cytochrome P450 isoforms, which are essential substances in the drug purification process.

Furthermore, sucralose alters the composition of the microbiome of the digestive tract, which ends up causing the reduction of the proportion of beneficial bacteria. In addition, if cooked at high temperatures, chloropropanol can form, which is a toxic compound. It can also alter the patient’s levels of glucose, insulin and glucagon-like peptide type 1 (GLP-1) [29]. 

### 3.7. Surfactants

#### Polysorbates 

Polysorbates are partial esters of sorbitol fatty acids and their copolymerized anhydrous with ethylene oxide. They are used as dispersant agents, emulgents, non-ionic sanitary surfactants, solubilizers, and moisturizers, among other things. 

In general, they are considered non-toxic and non-irritating. However, they have been associated with serious side effects, including deaths in under-weight neonates who received vitamin E preparations with this substance [25]. In addition, polysorbate 80 has been associated with increased mortality in new-borns [42].

### 3.8. Colorants

Colorants are excipients used to facilitate the identification of the formula by parents and patients. The most commonly used dyes are whip dyes, quinolones, triphenylmethane and xanthines.

Tartrazine (yellow number 5) has been implicated in anaphylactic reactions, edema, asthma, bronchospasm, eosinophils, angioedema and hives in patients with sensitivity to it. It appears to cause histamine degranulation of mast cells [29]. As a result, most global regulatory agencies restrict the use of dyes such as tartrazine, because azo dyes have been linked to hypersensitivity and ADHD reactions in children. These dyes can be replaced by plant dyes such as annatto, malt beta-carotene and turmeric and should not be used at all in paediatric formulations [28].

### 3.9. Excipients not Recommended in Paediatrics and Paediatric Formulations

To investigate the exposure of children to excipients not recommended at an early age, a compilation of paediatric formulations (nationally and internationally) was made (see Appendix C, Appendix D, Appendix E and Appendix F). As will be seen below, most of these formulations contain some excipient not recommended in paediatrics:

In Appendix C, there is a summary table (Table A2) of examples of solid and semi-solid medicines used in the paediatric population, marketed in Spain. Additionally, a list of excipients and relevant characteristics of the pharmaceutical form (PF) is shown (performed consultation of CIMA database, September 2020). It clearly shows that the reason such excipients are not recommended for the paediatric population is because of the adverse effects they may cause, which include:

- Approximately 100% of the formulations shown here carry at least one excipient not recommended for the paediatric population.
- Benzalkonium chloride, methyl para hydroxybenzoate and propyl para hydroxybenzoate are some of the most commonly used preservatives in solid and semi-solid formulations for paediatric use, even though they are considered to be potentially toxic in neonates.
- Sucrose, aspartame and mannitol are used as sweetener. 100% of the oral solid formulations collected in Table A4 carry at least one excipient of these: 40% of formulations carry mannitol and aspartame; 20% carry the 3 excipients; 20% sucrose and aspartame and the remaining 20% only sucrose.
- Propylene glycol is another excipient commonly used in solid formulations as a solvent, moisturizer and preservative. Caution should be exercised in children under 4 years of age and neonates, as propylene glycols, at high doses, may cause alterations in the Central Nervous System, in addition to other side effects discussed in the previous sections of this paper.
- Microcrystalline cellulose, methylcellulose and ethyl cellulose are one of the most commonly used excipients in solid formulations. They have no major side effects, but in high amounts they can cause a laxative effect.
- Most of the solid formulations collected in Table A2 use flavourings such as grape essence, lemon flavouring, caramel cream aroma or orange essence, in order to achieve a better palatability. The main drawback of their incorporation into paediatric formulations is that they usually have a complex and poorly known composition [49].
- Lanolin is an excipient used in pastes and ointments, which are frequently used in the paediatric population. This excipient may cause skin hypersensitivity reactions, which is why caution should be exercised in patients with known sensitivity issues [50].

Appendix D (Table A3) lists marketed liquid formulations suitable for the paediatric population. Liquid formulations are the most common in paediatrics because of their easy administration. The need for at least one liquid formulation of any drug indicated in the paediatric population is becoming increasingly noticeable. Not all active principles are soluble or stable in water. Therefore, excipients are used in liquid formulations to improve the solubility of certain active principles and/or increase their stability. The problem is that most excipients found in adult formulations should not be used in paediatrics. However, as shown in Table A3, there are a wide variety of marketed formulations indicated in paediatrics that contain these non-recommended excipients:

- Ethanol, sorbitol and propylene glycol, despite being contraindicated in paediatrics, especially ethanol, are still included in some paediatric formulations.
- The addition of non-recommended sweeteners, such as sucrose, sucralose or sodium saccharine, is also seen in these paediatric formulations.
- The addition of preservatives in paediatric formulations should be avoided as much as possible, and if necessary, in the least amount. Parabens are among the safest preservatives in paediatrics, yet others that are not recommended are still used (e.g., Table A3: sodium benzoate, benzoic acid and benzyl alcohol). Benzalkonium chloride, despite not being recommended for asthmatic patients, is used for the formulation of most eye drops, nasal drops and gothic drops.

Appendix E (Table A4) and Appendix F (Table A5) provide examples of FDA-registered drugs (liquids and solids) and liquid formulations in paediatric use research, respectively. Like the other examples provided, these medicinal products and liquid formulations contain at least one excipient not recommended for the paediatric population, such as propylene glycol, polysorbates, methyl or propyl para hydroxybenzoate, benzyl alcohol, benzoic acid, ethanol or sucralose, among others.

- Excipients not recommended for paediatric population are most commonly used in oral solutions and suspensions (referred to in Table A4 and Table A5, propylene glycol, benzoic acid, polyethylene glycol, polysorbate 80 and sodium benzoate).
- Like the other examples, there is also frequent use of sweeteners (fructose, sucrose, sucralose, aspartame and sodium saccharine).
- Benzalkonium chloride is one of the most commonly used preservatives in ophthalmic and nasal drops, as shown in Table A4. It is usually a safe excipient, but can cause serious adverse effects, such as bronchoconstriction in asthmatic patients, ototoxicity in erotic preparations or respiratory failure in infants who ingest this excipient, this adverse effect being the most severe.

## 4. Promising Pharmaceutical Form in the Paediatric Population: ODT and 3D Drug Printing

The development of Orally Disintegrating Tablets (ODT) has received greater interest among researchers and the pharmaceutical industry over the past decade. ODT tablets are designed to dissolve quickly upon contact with saliva, in the absence of additional water, compared to traditional tablets [51].

ODT tablets offer several advantages, combining the properties of solid and liquid formulations. They are quickly ingested when inserted into the tongue, eliminating the need to chew the tablet, swallow it intact or take it with water. Currently, they are a widely accepted form of dosing, especially for patients who have difficulty swallowing (paediatric and geriatric), and for the treatment of patients where therapeutic compliance is difficult [51,52].

As a result of the rapid disintegration of ODT tablets, the active substance comes into contact with taste buds, so a key aspect to consider in these formulations is palatability. It is necessary to mask the taste of bitter active ingredients in order to develop successful formulations. In the past, sweeteners and aromas were used as methods of flavour masking in dispersible or rapidly disaggregation tablets. However, these additives were not a sufficient means to completely mask the taste. Currently, with scientific and technological advances, different dosing alternatives are available to mask the taste, such as freeze-deriding, microencapsulation, fluid bed coating or coating in supercritical fluids [51].

It should be mentioned that there is an innovative tool for pharmaceutical pre-formulation of ODT tablets. This tool makes it possible to predict whether a disintegrating excipient or a mixture of excipient powder + active substance is suitable for obtaining an oral dispersible tablet by direct compression or not: the new model SeDeM-ODT [53].

The SeDeM-ODT model (based on the SeDeM expert system) indicates the ability of a powder to be compressed, providing the Good Compressibility and oral dispersibility Index (IGCB). This index is composed of six main factors which indicate whether a powder mixture has the ability to be compressed by direct compression. Furthermore, it indicates whether the tablets are suitable for formulation as oral dispersible tablets. Thus, the SeDeM-ODT model facilitates the selection of excipients with the appropriate properties to produce ODT tablets using direct compression technologies [53].

**Figure 1:**
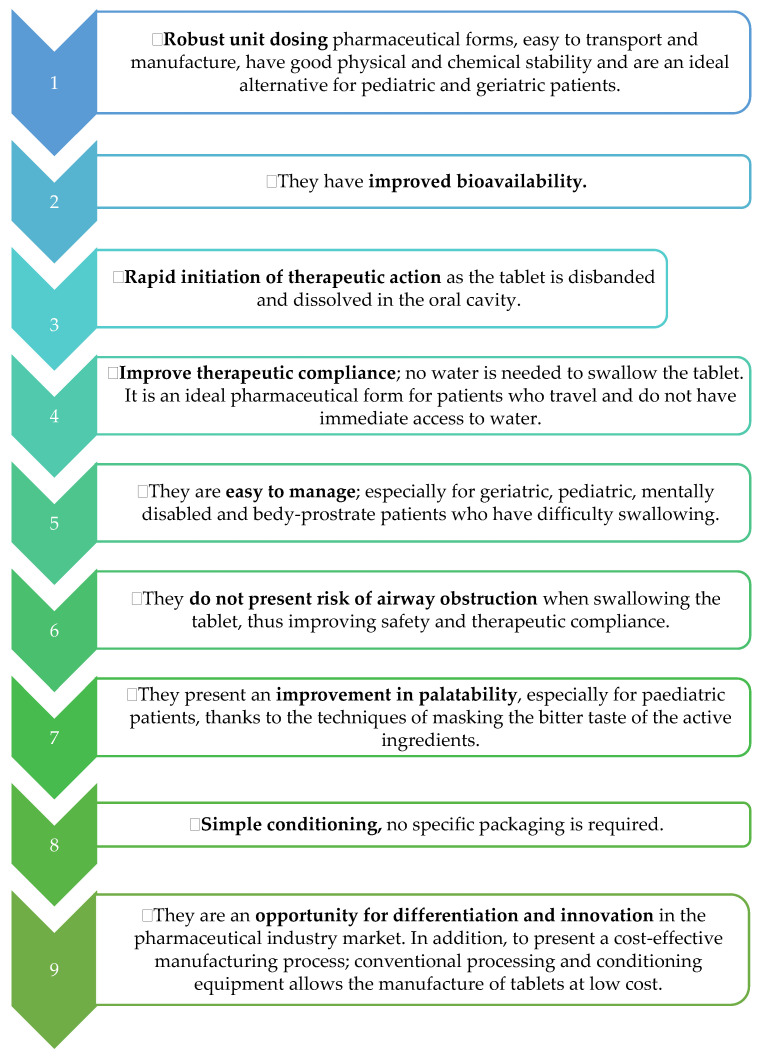
Characteristics and advantages of ODT tablets.

Figure 1 will detail several special features and advantages of ODT tablets [52,54].

**Figure 2:**
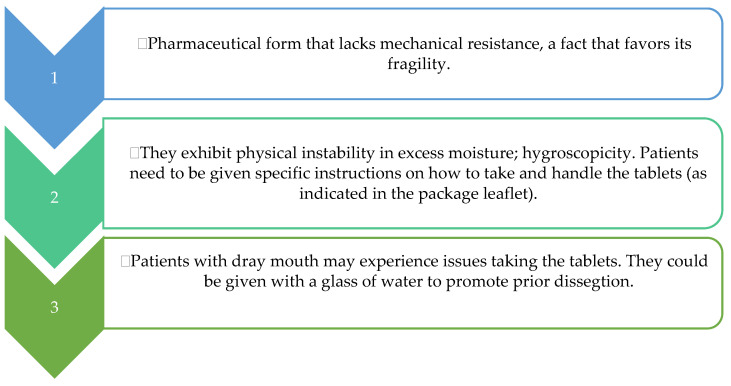
Disadvantages of ODT tablets.

Figure 2 specifies the most noteworthy drawbacks of ODT tablets [54].

On the other hand, the technical disadvantages associated with the manufacturing process of ODT tablets could be solved by three-dimensional drug printing technology. Generally speaking, this technology is supported by the following processes: a program capable of generating a file is required with the necessary information for printing the drug. This same program (also present on the computer that will control the printer) must be able to read the instructions contained in the generated file and convert it into precise commands for the 3D printer to generate the part [55].

The response to drugs may be different among patients, due to inter-individual variability, caused by both genetic and environmental factors. Accordingly, “patient-specific” or “tailor-made” dosage concepts could be an alternative to mass production in the traditional pharmaceutical industry. In this approach, 3D printing has proven to be a manufacturing technique with great potential, as it allows the creation of three-dimensional objects, layer by layer, with total freedom of form and design. Thus, obtaining customized pharmaceutical forms is one of the main objectives of 3D printing in the pharmaceutical sector [55].

Paediatric patients are one of the population groups with the greatest need for personalized dosing adapted to their requirements (age, weight, pathological status, etc.). However, most 3D printed drugs are solid oral formulations, which are not suitable for this population group. Medicinal gummies developed through 3D printing (tailor-made to the patient) could be a form of oral dosing suitable for paediatric patients, due to their striking appearance and pleasant organoleptic characteristics [55].

New advances in obtaining medicines and medical devices, using 3D printing technology, have generated novel perspectives in the processes of obtaining these products. At present; however, several issues are perceived that will need to be resolved as the perfection and implementation of this technique progresses, in order to make it a common process of obtaining medicines and medical devices.

## 5. Conclusions

The critical study suggests that excipients are often used at higher concentrations than recommended in international paediatric guidelines, and with inappropriate labelling, increasing the potential risks associated with the various excipients discussed [26]. 

Indeed, the pharmacokinetic and pharmacodynamic profiles of the child population vary substantially, with paediatric safety profiles related to the age and development of excipients often differing from those of adults [48]. The most toxic excipients in neonates are known to be sodium benzoate, propylene glycol, methyl para hydroxybenzoate, propyl, sodium saccharine, benzyl alcohol, benzalkonium chloride, polysorbate 80 and ethanol [56]. However, these excipients are used in formulations according to the study conducted.

European new-borns receive several potentially harmful pharmaceutical excipients: parabens, polysorbate 80, propylene glycol, benzoates, sodium saccharine, sorbitol, ethanol and benzalkonium chloride. According to the study conducted by Nellis and collaborators [36], there are regional variations in the neonatal administration of these potentially harmful excipients. This suggests the possibility of reducing exposure to parabens, polysorbate 80, propylene glycol and sodium saccharine by replacing it with products without these excipients. However, a joint effort by the regulatory authorities on medicines, in particular the paediatric committees, will be necessary. Current therapeutic options for the paediatric population justify further toxicokinetic and drug safety studies so that they are tailored to the special needs of the paediatric population.

In general, there is little information regarding excipients in paediatrics. It is of the utmost importance to develop new research related to the safety and toxicity of excipients to reduce the prevalence of adverse effects in paediatric populations. Gallon formulators can formulate safer, more stable and higher quality products. Furthermore, the possible adverse effects of the active ingredients and the excipients used in the paediatric population should be reconsidered—since excipients that are safe in adults—may have potentially toxic effects in children.

Finally, the development of databases such as STEP is relevant and beneficial for the development and use of drugs in paediatrics. Additionally, the SEEN project is relevant both nationally and internationally, as it reveals the current status of excipients and takes into account the frequency and quantity (in terms of medicines given to new-borns and young children).

## Figures and Tables

**Table 1:** Toxicity databases and public resources.

Name	Website	Creator
ACToR —Aggregated Computational Toxicology Resource	www.actor.epa.gov/actor/home.xhtml (accessed on 15 November 2020)	US Environmental Protection Agency’s (EPA) National Center for Computational Toxicology (NCCT)
STEP—Safety and Toxicity of Excipients for Paediatrics *	www.eupfi.org/step-database-info/ (accessed on 15 November 2020)	European Paediatric Formulation Initiative
TOXNET—Toxicology Data Network	www.nlm.nih.gov/toxnet/index.html (accessed on 15 November 2020)	Specialized Information Services (SIS) USA
Vitic	www.lhasalimited.org/products/vitic.htm (accessed on 2 November 2020)	Lhasa Limited

* The purposes of the STEP database can be consulted in the Appendix A.

## Data Availability

The data presented in this study are available on the Reference list.

## Appendix A. Purposes of the STEP Database

More specifically, the purposes of the STEP database are [22] to:

1. Serve as a public base for evidence regarding the safety and toxicity of excipients in order to allow the pharmaceutical industry, academics, pharmacists, physicians and regulators to make informed decisions.
2. Improve prospects of identifying potential security issues in the early stages of the development process when excipients are selected.
3. Help highlight any relationships between exposure and evidence of clinically significant toxicity in the paediatric age group in general, or in paediatric subpopulations.
4. Identify possible differences in expression, types or patterns of toxicity in children compared to adults. Provide a basis for assessing the need to generate new data for paediatric medicines (e.g., bridge studies, juvenile toxicity studies, etc.), in order to clarify what kind of new data, knowledge gaps or studies may be needed.
5. Support companies with their regulatory presentations with easily available information.
6. Support and improve research activities by providing a platform to share unreleased data and available data with corporate entities.

## Appendix B

**Table A1:** Most important characteristics of the excipients discussed in this paper (in alphabetical order).

Excipient	Functions	DAI *	Recommendations	Adverse Effects	References
Aspartame	Artificial Sweetener	40 mg/kg	- Contraindicated in patients with phenylketonuria	- Neurological involvement: neurotoxicity, epilepsy, headache, panic attack, hallucinations - Hypersensitivity reactions: vascular, granulomatous panniculitis - Cross reaction with sulfamides	[29,35]
Benzalkonium chloride	Preservative	NA	- Caution in asthmatic patients	- Bronchoconstriction - Ototoxicity - Hypersensitivity	[29]
Benzyl alcohol	Preservative	5 mg/kg	- Contraindicated in children under 3 years of age by immature their metabolism	In new-borns and children under 3 years of age cause: - Metabolic acidosis and respiratory depression - Intraventricular haemorrhage - Cerebral palsy and developmental delay - Hypersensitivity reactions	[29,35,36,39,40,41,42,43,44]
Ethyl alcohol	Solvent and preservative	6 mg/kg/dose (<6 years)	Paediatric formulations should not exceed the following limits of ethanol: - In children over 12 years of age: less than 10% (*v/v*) - In children 6–12 years old: less than 5% (*v/v*) - In children under 6 years of age: less than 0.5 (*v/v*)	- Hypoglycaemia, acidosis and hydroelectrolytic alterations - Stupor, coma respiratory and CNS depression, cardiovascular toxicity	[17,26,27,29,31,32]
Glycerol	Solvent, sweetener, viscosizer and preservative	10 g/dose	- Caution in paediatric population - Do not exceed the safe daily dose (1.0–1.5 g/kg body weight)	- Mucositis in the stomach - Diarrhoea and electrolyte disturbances	[1,29]
Lactose	Diluent	NA	- Caution in patients with lactose intolerance - Contraindicated in galactosemia	Symptoms of lactose intolerance: severe abdominal pain, flatulence, bloating or swelling and diarrhoea. - Systemic symptoms such as muscle and joint pain and eczema - In children it can cause dehydration, bacterial proliferation and metabolic acidosis	[1,28]
Parabens	Preservative	10 mg/kg	- It is recommended to avoid its use in neonates	- Cross hypersensitivity reactions in patients allergic to acetylsalicylic acid - Hyperbilirubinemia in new-borns	[29,35,36]
Phthalates	Coating agents (plasticizers)	NA	- Not recommended for use in pregnant women or children under 3 years of age	- Anomalies in the development of the foetus: cleft palate and skeletal malformations. May lead to stillbirth	[28]
Polyethylene glycol	Solvent, suspensor and viscosity agent	10 mg/kg	- Caution in new-borns and infants	- Nephrotoxicity - Gastrointestinal disorders - Laxative effect	[1,28]
Polysorbates	Dispersing, emulgent, surfactants, solubilizing and moisturizing agents	NA	- Caution in new-borns	- Serious adverse effects: deaths in low-weight neonates who received vitamin E preparations with polysorbates. - Polysorbate 80: increased mortality in new-borns	[25,42]
Propyl gallate	Antioxidant	NA	- Caution in new-borns	- In neonates it can cause dermatitis, skin allergy and methemoglobinemia	[29]
Propylene glycol	Solvent, moisturizing and preservative	- Neonates: 1 mg/kg - Under 5 years: 50 mg/kg - Adults: 500 mg/kg	- It is recommended to avoid in children under 4 years of age because of lack of metabolic maturation	- CNS depression - Laxative effect from high osmolality after oral administration	[29,33,34]
Saccharine	Sweetener	2.5 mg/kg	- It is recommended to limit the daily dose in pregnant women and children	- Urticaria, itching and eczema - Photosensitization - GI disturbances: Nausea and diarrhoea	[29,48]
Sorbitol	Sweetener and diluent	- Children 0–2 years 5 mg/kg - Over 2 years: 140 mg/kg	- Contraindicated in patients with fructose intolerance - Not recommended for use in patients with hypoglycaemia	- Gastrointestinal disorders - It can cause hepatic damage with comma and even death	[28,29,30]
Starch	Diluent and added	NA	- Conservation in dry environment - Well tolerated by children	- In case of moisture, carcinogenic aflatoxins may occur	[29]
Sucralose	Sweetener	15 mg/kg	- Caution in patients with metabolic disorders	Alters the composition of the digestive tract microbiome - At high temperatures chloropropanol may form - May alter glucose, insulin and GLP-1 *^2^ levels	[29]
Sucrose	Sweetener	NA	- Not recommended for use in children with type I diabetes	- Dental damage - At very high doses on a daily basis I could be carcinogenic	[29]
Sulphites	Antioxidant	NA	- Avoid in asthmatic patients	- Hypersensitivity and bronchospasm reactions	[29]
Tartrazine, quinolines, triphenylmethane, xanthines	Colorants	NA	- It is recommended not to use them in paediatric formulations	- Hypersensitivity reactions in patients’ sensitive to tartrazine - Azo colorants: cross-sensitivity reactions with acetylsalicylic acid Erythromycin: photosensitization reactions	[28,29]
Thiomersal	Preservative	NA	- Avoid use in vaccines as a preservative due to its side effects	- Hypersensitivity reactions - Autism spectrum disorders	[28,29]

* ADI: Admissible Daily Intake; *^2^ GLP-1: Glucagon Like Peptide; NA: Not Available.

## Appendix C

**Table A2:** Examples of solid and semisolid medicines used in Spain for paediatric population: List of excipients and relevant characteristics of FF (Performed consultation of CIMA database, September 2020).

Pharmaceutical Form			Excipients	API	Pharmaceutical Form Characteristics	References
SOLID PREPARATIONS	POWDERS	Example 1: Amoxicillin Normon 250 mg/5 mL EFG Oral Suspension Powder	Saccharose, Glucose, Methyl parahydroxybenzoate (E-218), Propyl parahydroxybenzoate (E-216), Anhydrous sodium citrate, Colloidal silica and Orange essence	Amoxicillin	- Powders are administered after prior dissolution. - They are little employees in the paediatric population; present the drawback that it is difficult to mask the bad taste. - Risk of accidental aspirations. - They are usually used in master formulation and for the administration of antacids.	[29,57,58]
		Example 2: Azithromycin Sandoz 200 mg/5 mL EFG Oral Suspension Powder	Sucrose, Xanthan gum (E415), Hydroxypropyl cellulose, Anhydrous trisodium phosphate, Colloidal anhydrous silica (E551), Aspartame (E951), Aroma of caramel cream and Titanium dioxide (E171)	Azithromycin		
	GRANULATED	Example 1: Paediatric Gelocatil 325 mg Granules	Calcium carbonate, Sodium hydrogen carbonate, Citric acid anhydrous, Anhydrous sodium citrate, Aspartame (E-951), Sucrose, Mannitol (E-421), Amorphous silica, Glycerol die-stearate type 1, Croscarmellose sodium, Sodium glycolate starch type A (potato starch) gluten-free, Ethyl cellulose, Hydroxypropyl methylcellulose and Polyethylene glycol 400	Paracetamol	- Granules are more stable and fluid than powders. - The most used are effervescent granules, which in the presence of water react by releasing carbon dioxide, which protects the stomach and partly anesthetizes taste buds. - They should be completely dissolved prior to administration in order to reduce bicarbonate intake. - Children are often pleased by their resemblance to certain refreshing drinks.	[29,59]
SOLID PREPARATIONS	ORAL DISPERSIBLE TABLETS (ODT)	Example 1: Apiretal 325 mg oral dispersible tablets	Ethyl cellulose, Microcrystalline cellulose, Crospovidone, Aspartame (E-951), Colloidal silica, Mannitol, Talco, Magnesium stearate and Grape essence	Paracetamol	As advantages of oral dispersible tablets, the following stand out: - They combine the advantages of liquid forms and solid oral forms. - An exact dose may be given compared to liquids. - They have a pleasant taste, thus facilitating therapeutic compliance in the paediatric population. - No need to swallow the tablet or drink water; dissolves rapidly in saliva, being an appropriate choice for patients with swallowing problems, such as children or geriatric patients. - They are safe and effective and can be bio-equivalent with respect to conventional tablets. - They have rapid absorption and, therefore, a rapid introduction of the therapeutic effect. Disadvantages include: - The lack of mechanical resistance presented by traditional tablets. - The possibility of physical instability in excess moisture. - ODTs require special conditioning to ensure their stability.	[54,60,61]
		Example 2: Junifen 200 mg lemon-flavored oral dispersible tablets	Ethyl cellulose, Precipitated silicon dioxide, Hypromellose, Mannitol, Aspartame (E-951), Croscarmellose sodium, Magnesium stearate and Lemon flavouring	Ibuprofen		
SOLID PREPARATIONS	SUPPOSITORIES	Example 1: Febectal Infants 150 mg Suppositories	Colloidal anhydrous silica, Solid semi-synthetic glycerides	Paracetamol	As advantages, the following stand out: - Generally, they avoid gastric intolerance problems. - They are of interest when the medicine is inactive orally, the patient is unconscious or are children who refuse to swallow the medication. - They avoid inactivation by the effect of first liver step. Disadvantages include: - Reproducible behaviour can only be obtained if absorbed into an area two centimetres from the end of the rectum. - Absorption of the active substance may be erratic. - As it avoids the effect of first liver step, it can increase the possibility of poisoning. - In certain cultures, it is a form that is not well accepted socially.	[29,62]
SEMI-SOLID PREPARATIONS	GELS	Example 1: Fenistil 1 mg/g Gel	Benzalkonium chloride, Disodium edetate, Carbomer, Sodium hydroxide, Propylene glycol amd Purified water	Dimethindene maleate	- It is a semi-soft transparent colloid, with a large proportion of liquids. - Low penetration power. - Many incompatibilities with active substances. - It is easy to apply, pleasant and soothing for its refreshing properties.	[29,63]
SEMI-SOLID PREPARATIONS	CREAMS	Example 1: Perme-Cure 5% Cream	Butylhydroxytoluene (E-321), Castor Oil, Deionized water, Steareth-2, Ceteareth-2-Phosphate, Sosa to the 20 %, Vitamin E acetate, Phenonip, Citric acid, Disodium edetate and Scent	Permethrin cis:trans (25:75)	As advantages, the following stand out: - Comfortable and easy application. - Provide a controlled release of the active substance. - They act as emollients and moisturizers, due to their composition	[64]
	OINTMENTS	Example 1: Oftacilox 3 mg/g Ophthalmic Ointment	Liquid paraffin and White Vaseline	Ciprofloxacin	- Ointments are forms of external use intended to be administered by gentle friction on a surface of the body, to achieve a local action or with the aim of penetrating the drug through it. - In many cases, the topical route is a route of absorption comparable to oral or other, so the dosage and duration of treatment must be very well specified. New-borns and infants have a very increased skin-to-weight ratio. Coupled with the fact that at this age the skin is very permeable, it makes them especially vulnerable to toxic frames by ointments.	[29,65]
	PASTES	Example 1: Anti-congestive Cusi (Paste Lassar)	Lanolin (wool fat), Liquid Vaseline and Stringy Vaseline	- Zinc oxide - Corn starch	- This is a suspended ointment. - They are used when you want to locate the action of the active substance to a specific area, as they are irritating and staining.	[29,66]
SEMI-SOLID PREPARATIONS	NON-CREAM EMULSIONS	Example 1: Lactisona 10 mg/mL Skin Emulsion	Carbomer 940, 1,3-dimethylol-5,5-dimethyl hydantoin, Dihydro-acetic acid, Pyrrolidone sodium carboxylate, Lactic acid, Sodium hydroxide, Stearyl alcohol, Glycerol stearate, Cetyl alcohol, Isopropyl palmitate, Mineral oil, Myristyl lactate, Fragrance and Water	Hydrocortisone	- Emulsions are a dispersed system, stabilized by the addition of an adequate emulsifier, two immiscible phases, where both the internal and external phases are liquid. - The emulsions enable fat-soluble and water-soluble active ingredients to come into contact with the skin simultaneously, encompassing each of them in the phase of the emulsion for which they have the greatest affinity. - Patients or users of topical application preparations often prefer emulsion vehicles to those of any kind.	[50,67]

## Appendix D

**Table A3:** Examples of liquid medicines used in paediatrics: List of excipients and relevant characteristics of Pharmaceutical Form.

Pharmaceutical Form			Excipients	API	Pharmaceutical Form Characteristics	References
LIQUID PREPARATIONS	ORAL SOLUTIONS	Example 1: Diazepam 2 mg/5 mL Solution without sugar	Sodium Docusate, Aluminium silicate and Magnesium, Propylene glycol, Raspberry Flavour, Sodium Saccharine, Precool Erythrosine (E127), Sorbic Acid (E200), Propyl para hydroxybenzoate, Methyl para hydroxybenzoate, Sorbitol, Liquid (Non-Crystalized) (E420) and Glycerol (E422)	Diazepam	As advantages, the following stand out: - Release of the active substance(s) much faster than in solid forms. - The dosages are correctly expressed in milligrams, micrograms and U/mL, allowing them to be adapted to the child’s weight. - Easy and comfortable dosing, as it is in volume (spoons, drops, etc.) - Less irritation effect if it is an aggressive medicine, at the gastric level, as it is dampened by dilution. - Solutions, suspensions or emulsions are obtained, depending on the size of the particles of the internal phase. As disadvantages they present:	[29,49,68,69,70]
		Example 2: Paracetamol Level 100 mg/mL Oral Solution	Citric acid, Sodium hydroxide, Sucrose, Propylene glycol, Macrogol, Strawberry Essence, Cochineal Red A (Ponceau 4R) (E-124), Hydrochloric Acid 5 N and Purified Water	Paracetamol		
		Example 3: Diazepam Intensol™ Oral Solution 5 mg/mL * *Do not use in children under 6 months of age*	Alcohol, Yellow D&C 10, Polyethylene glycol, Succinic Acid and Water	Diazepam	- Greater ease and possibility of contamination than solid pharmaceutical forms, which forces the addition of preservatives.	[71,72]
		Example 4: Prednisolone 10 mg/mL Oral Solution	Sodium Methyl para hydroxybenzoate, Sodium Propyl para hydroxybenzoate, Glycerol, Sodium Saccharine, Sodium Edetate, Sodium Aqueous solutions of medicinal substances that areDihydrate, Orange flavour (contains propylene glycol), Sodium hydroxide and Purified Water	Prednisolone		
LIQUID PREPARATIONS	ORAL SOLUTIONS	Example 5: Ozalin	Citric acid monohydrate, Gamma-cyclodextrin, Sucralose, Orange flavour (contains 70–80% ethanol), Sodium hydroxide, injectable water	Midazolam	*See “Pharmaceutical Form Characteristics (Oral Solutions)” section of the previous page*	[73,74,75]
		Example 6: Flumil 20 mg/mL Oral Solution	Para-Hydroxybenzoate Methyl (E218), Sodium Benzoate (E211), Sodium Edetate, Carmellose Sodium, Sodium saccharine, Sodium Cyclamate, Sucralose, Raspberry Aroma, Sodium Hydroxide and Purified Water	Acetyl cysteine		
		Example 7: Paediatric Lanacordin 0.05 mg/mL * *Including newborns and premature*	Sucrose, Ethanol, Tartrazine (E-102), Anhydrous Sodium Phosphate, Citric Acid (E-330), Methyl Hydroxybenzoate, Lime Essential Oil, Propylene glycol (E-1520) and Purified Water	Digoxin		
LIQUID PREPARATIONS	ORAL SUSPENSIONS	Example 1: Paracetamol 120 mg/5 mL Oral Suspension	Propylene glycol, Methyl Hydroxybenzoate, Propyl Hydroxybenzoate, Xanthan Gum, 70% Sorbitol Solution, Sucrose, Mango flavour and Purified Water	Paracetamol	As advantages, the following stand out: - Suspensions are the ideal pharmaceutical forms for the administration of non-water-soluble active ingredients. - The fact that the active substance is insoluble, allows an extension of the time of action in the body. - It is easier to mask the taste than in syrups and elixirs (more pleasant for children). - Good relative bioavailability.	[76,77]
		Example 2: Junior Parapaed 120 mg/5 mL Oral Suspension	Ethanol, Polysorbate 80, Glycerol, Magnesium and Aluminium silicate, Liquid maltitol syrup, Sodium saccharine (E954), xanthan gum, cherry flavour, sodium benzoate, Citric acid monohydrate and purified water	Paracetamol		
LIQUID PREPARATIONS	ORAL SUSPENSIONS	Example 3: Mycostatin 100.000 UI/mL Oral Suspension	Sucrose, 96% ethanol, Carmellose sodium, Cinnamic aldehyde, Mint Essence, Cherry Aroma, Anhydrous Disodium Hydrogen phosphate, Glycerol (E-422), Methyl para hydroxybenzoate, Propyl para hydroxybenzoate, Sodium Hydroxide, Hydrochloric Acid and Purified Water	Nystatin	Disadvantages include: - Sediment formation. - Difficulty removing the viscosity of the vehicle. - Less stability than solid shapes, solutions and emulsions. - The use of very fine particle size causes the formation of sediments that are very difficult to re-suspend. *It is important to shake the suspension for at least 10 s before use*	[78,79]
		Example 4: Paediatric Algidrin 20 mg/mL Oral Suspension * *Do not give to children under 3 months of age*	Microcrystalline cellulose, Carboxymethylcellulose sodium, Sorbitol (E-420), Maltitol (E-965), Beta-cyclodextrin, Sodium Saccharine, Sucralose (E-955), Forest Fruit Aroma, Allura AC Red Colouring (E-129), Methyl para hydroxybenzoate, Ethyl para hydroxybenzoate, Propyl para hydroxybenzoate and Purified Water	Ibuprofen (Lysine)		
LIQUID PREPARA-TIONS	ORAL SUSPEN-SIONS	Example 5: Paediatric Septrin 8 mg/40 mg/mL Oral Suspension ** Suitable for infants from 6 weeks of age*	Sorbitol, Glycerol (E-422), Dispersible Cellulose, Carmellose Sodium, Polysorbate 80, Methyl para hydroxybenzoate, Sodium Benzoate, Sodium Saccharine, Banana flavour (Propylene Glycol E-1520, Sodium Citrates E-331), Ethanol 96°, Vanilla flavour (Benzyl Alcohol, Caramel Colour E-150d, Propylene Glycol E-1520, Glycerol E-422, Water), Purified Water.	- Trimetho-prim - Sulfametho-xazole	*See “Pharmaceutical Form Characteristics (Oral Suspension)” section of the previous page*	[80]
LIQUID PREPARATIONS	ELIXIRS	Example 1: Paracetamol Elixir Pediátrico 120 mg/5 mL	Ethanol 96° (10% *v*/*v*), Propylene glycol, Inverted Syrup, Amaranth Solution (E123), Glycerol, Glycerine, Chloroform and Concentrated Raspberry Juice	Paracetamol	- Hydro alcoholic solution sweetened with low sugar. - It has high alcohol content, which will have to be considered at certain ages, as it can create addition or generate other side effects: drowsiness and various dangers arising.	[29,81,82]
		Example 2: Lanoxin Elixir ** Fit for premature neonates*	Methyl Hydroxybenzoate, Sucrose, Sodium Phosphate Anhydrous, Citric Acid Monohydrate, Quinine Yellow, Ethanol (96%), Propylene Glycol, Lime flavour and Purified Water	Digoxin		
	SYRUPS	Example 1: Daleron Syrup 120 mg/5 mL	Sorbitol, Glycerol, Xanthan Gum, Maltitol, Microcrystalline Cellulose, Croscarmellose Sodium, Sodium Benzoate, Citric Acid, Pineapple flavour, Riboflavin and Purified Water	Paracetamol	- Syrups are liquid solutions with sweetening, flavouring and viscosizing properties. They are almost saturated aqueous solutions of sucrose (64%). They have the following drawbacks: - Alterations that require the incorporation of preservatives and specify	[29,83,84]
		Example 2: Loratadine 5 mg/mL Syrup Oral Solution	Propylene glycol, Glycerol, Sodium Benzoate, Citric Acid Monohydrate, Sucrose, Peach flavour and Purified Water	Loratadine		
LIQUID PREPARA-TIONS	SYRUPS	Example 3: Polaramine 0.4 mg/mL Syrup ** Not suitable for children under 2 years old*	Ethanol, Sucrose, Sodium Citrate, Sodium Chloride, Sorbitol, Methyl paraben, Propyl paraben, Menthol, Apricot flavour, Orange flavour, Ponceau 4R Colouring (E-124) and Purified Water	Dexchlorpheni-ramine maleate	See “Pharmaceutical Form Characteristics (Syrups)” section of the previous page	[85,86]
		Example 4: Paediatric Mucosan 3 mg/mL Syrup	Hydroxyethyl cellulose, Sucralose, Benzoic Acid (E-210), Wild Berry Aroma, Vanilla Aroma and Purified Water	Ambroxol hydrochloride		
LIQUID PREPARATIONS	ORAL DROPS IN SOLUTION	Example 1: Romillary 15 mg/mL Oral drops in Solution * Not recommended for use in children under 2 years of age	Propylene glycol, anhydrous ethanol, Flavourings: coriander oil, orange essential oil and lemon tetraroma, macrogol glycerol ricinolate (chromophore EL), Methyl para hydroxybenzoate, Propyl para hydroxybenzoate, sodium saccharine, citric acid monohydrate, sodium hydroxide and purified water	Hydrobromide dextromethorphan	- Oral liquid medicinal products may be placed on the market in the form of drops for children of different ages. - The main benefits of drops are low dosing volume, facilitating swallowing and dosing flexibility. As disadvantages, the following stand out: the variation of the droplet size and errors in the count, which would result in an incorrect dosage. This can cause serious problems in those medicines with a narrow therapeutic margin.	[87,88,89,90]
		Example 2: Alerlisin 10 mg/mL Oral Drops in Solution ** Do not use in children under 2 years of age*	Glycerol, Propylene glycol (E-1520), Sodium Saccharine, Methyl para hydroxybenzoate, Propyl para hydroxybenzoate, Sodium Acetate, Glacial Acetic Acid and Purified Water	Cetirizine hydrochloride		
LIQUIDPREPARA-TIONS	ORAL DROPS IN SOLUTION	Example 3: Paediatric Cleboril 62.5 g Oral Drops in Solution	Benzoic acid (E-210), Sodium hydroxide and purified water	Clebopride malate	See “Pharmaceutical Form Characteristics (Oral Drops in Solution)” section of the previous page	[91,92,93]
		Example 4: Fluor Lacer 1.4 mg/mL Oral Drops * Indicated for tooth decay prophylaxis in children 1–6 years old	Sodium Saccharine, Propylene glycol, Methyl para hydroxybenzoate, Propyl para hydroxybenzoate, Disodium edetate, Cochineal Red Colouring (E-124), Strawberry Aroma and Purified Water	Sodium Fluoride		
		Example 5: Hydropolivit Oral Drops in Solution ** Recommended for children over 2 years old*	Propylene glycol, Polysorbate 80, Sorbitol 70% (E-420), Glycerol (E-422), Sodium Saccharine, Sodium Edetate, Monothioglycerol, Methyl para hydroxybenzoate, Butylhydroxyanisole (E-320), Banana Essence, Vanilla Essence, Sodium Hydroxide and Purified Water	-Retinol palmitate Cholecalciferol Alpha-tocopherol acetate Riboflavin Pyridoxine hydrochloride Ascorbic acid Biotin Nicotinamide		
LIQUID PREPARATIONS	ORAL DROPS INSUSPENSION	Example 1: Zamene 22.75 mg/mL Oral Drops in Suspension * Special interest in paediatrics. Not recommended in children under 2 months of age.	Aluminium and Magnesium silicate, Carboxymethylcellulose sodium, Benzyl alcohol, 70% Sorbitol, Polysorbate 80, Acetic Acid and Purified Water	Deflazacort	They have the same characteristics as oral drops in solution	[94,95]
		Example 2: Dezacor 22.75 mg/mL Oral Drops in Suspension * Special interest in paediatrics. Not recommended in children under 2 months of age.	Sorbitol solution 70%, Carboxymethylcellulose sodium, Aluminium silicate and magnesium, Polysorbate 80, Benzyl Alcohol, Sucralose, Tropical Fruit Aroma, Citric Acid Monohydrate, Sodium Hydroxide and Purified Water	Deflazacort		
	OPHTHALMIC DROPS OR COLLYRIUMS	Example 1: Atropine BP 1.0% (*w*/*v*)/Vistatropin 1.0% (*w*/*v*) Eye drops in solution	Benzalkonium chloride in solution and purified water	Atropine sulphate	- Sterile solutions aimed at exercising their action in the conjunctiva. - May cause systemic side effects, especially observed after instillation of mydriatic eye drops.	[29,68,96,97]
		Example 2: Chibroxin 3 mg/mL Collyrium in solution	Sodium Acetate, Benzalkonium Chloride, Disodium Edetate, Concentrated Hydrochloric Acid, Sodium Chloride and Water for Injections	Norfloxacin		
LIQUID PREPARATIONS	NASAL DROPS	Example 1: Rhinovin^®^ Children’s 0.5 mg/mL Nasal Drops in Solution ** Do not use in children under 6 years of age*	Dihydrogen phosphate of sodium dihydrate, disodium phosphate dodecahydrate, disodium Edetate, Benzalkonium Chloride, Sorbitol (E420), Hypromellose, Sodium Chloride and Purified Water	Xylometazoline hydrochloride	- Aqueous solutions of medicinal substances that are instilled through the nose and act on the nasal mucosa. - Oils are contraindicated in their formulation, because the ciliary function has to be maintained. - It can be an excellent route of systemic administration, in addition to use as a topical route (there are promising studies with insulin and other substances).	[29,68,98,99]
		Example 2: Utabon Children 0.25 mg/mL Nasal Drops in Solution ** Do not use in children under 6 years of age*	Benzalkonium chloride, anhydrous disodium hydrogen phosphate, Sodium dihydrogen phosphate dihydrate, glycine (E-640), Sorbitol (E-420) and Purified water	Oxymetazoline hydrochloride		
	OTIC DROPS	Example 1: Otic cetraxal 3 mg/mL Otic drops en Solución ** Indicated in adults and child*	Lactic acid, Povidone, Anhydrous Glucose, Propylene glycol, Methyl para hydroxybenzoate, Propyl para hydroxybenzoate, Hydrochloric Acid and Purified Water	Ciprofloxacin	- Liquid preparations to apply to the middle and outer ear. - The active substances are usually antiseptics, local anaesthetics and antibiotics. Excipients have to be suitable to achieve a pH of 5–6.	[29,100,101]
		Example 2: Otix Otic Drops in Solution * Do not administer in children under 2 years of age	Benzalkonium Chloride, Sulphuric acid, Sodium Chloride, Sodium Hydroxide, Tribasic Sodium Citrate, Polysorbate 80, Citric Acid and Purified Water	- Dexamethasone sodium phosphate - Trimethoprim - Polymyxin B sulphate		
LIQUID PREPARATIONS	OTIC DROPS	Example 3: Ciproxin Simple 3 mg/mL Otic Drops in Solution * Not recommended for children under 1 year old	Benzalkonium Chloride, Sodium Acetate Trihydrate, Glacial Acetic Acid, Mannitol (E-421), Disodium Edetate, Hydrochloric Acid and/or Sodium Hydroxide and Purified Water	Ciprofloxacin hydrochloride	See “Pharmaceutical Form Characteristics (Otic Drops)” section of the previous page	[102]
PARENTERAL PREPARATIONS FOR INJECTION	INTRAVENOUS	Example 1: Digoxin Kern Pharma 0.25 mg/mL solution for injection * including premature neonates	Ethanol, Propylene Glycol, Citric Acid Anhydrous, Bi-sodium Anhydrous Phosphate and Bi-distillate Water.	Digoxin	- The intravenous line is the one of choice in new-borns and in emergencies. It achieves a quick effect and are easy to dos. - Risk of infection and can be painful at times and cause difficult-to-resolve injuries.	[29,103]

## Appendix E

**Table A4:** Examples of FDA-registered drugs used in paediatrics (FDA and DAILYMED database consultation October 2020).

Pharmaceutical Form			Excipients	Active Principle	Age	References
LIQUID PREPARATIONS	ORAL SOLUTIONS	Abilify Solution Oral	Disodium edetate, fructose (200 mg per mL), glycerine, dl-lactic acid, methylparaben, propylene glycol, propylparaben, sodium hydroxide, sucrose (400 mg per mL), and purified water. The Oral solution is flavoured with natural orange cream and other natural flavours	Aripiprazole	6 to 18 years	[104]
		Demerol Solution Oral	Benzoic acid, flavour, liquid glucose, purified water, saccharin sodium	Meperidine hydrochloride	Adult and paediatric patients	[105]
		Diazepam Oral Solution (Lannett Company)	Polyethylene glycol, propylene glycol, non-crystallizing sorbitol solution, sodium citrate anhydrous, bitterness modifier flavour, anhydrous citric acid, peppermint flavour, mint flavour, FD&C Network No. 40 aluminium lake, D&C Yellow No. 10 aluminium lake and purified water	Diazepam (5 mg/5 mL)	Children from 6 months	[106]
	ORAL SUSPENSIONS	Adzenys ER (Extend release)	Purified water, sorbitol, propylene glycol, xanthan gum, natural orange flavour, methacrylic acid and methyl methacrylate copolymer, sodium polystyrene sulfonate, vegetable oil, triethyl citrate, methylparaben, citric acid, sucralose, propylparaben, orange colour (FD&C Yellow No. 6), and polyethylene glycol	Amphetamine	6 to 17 years	[107]
		Children’s Tylenol^®^ Cold + Cough + Sore Throat Oral Suspension	Anhydrous citric acid, D&C network No. 33, FD&C network No. 40, flavours, glycerine, microcrystalline cellulose and sodium carboxymethyl cellulose, purified water, sodium benzoate, sorbitol solution, sucralose, xanthan gum	Acetaminophen 160 mg Dextromethorphan hydrobromide 5 mg	4 to 11 years	[108]
	ORAL SUSPENSIONS	Dyanavel XR (Extend release)	Anhydrous citric acid, bubble-gum flavour, glycerine, methylparaben, modified food starch, polysorbate 80, povidone, polyvinyl acetate, propylparaben, sodium lauryl sulphate, sodium polystyrene sulfonate, sucralose, triacetin and xanthan gum	Amphetamine	Children from 6 years	[109]
	SYRUPS	Midazolam hydrochloride syrup	Anhydrous Citric Acid, D&C Network No. 33, edetate disodium, glycerine, sodium benzoate, sorbitol, Water, Hydrochloric Acid, Sodium Citrate	Midazolam hydrochloride	Children from 6 months	[110]
LIQUID PREPARATIONS	OTIC DROPS	Ciprofloxacin and dexamethasone suspension/drops	Benzalkonium chloride, boric acid, edetate disodium, acetic acid, sodium acetate, sodium chloride, sodium hydroxide, tyloxapol, water, hydrochloric acid, hydroxyethyl cellulose (3000 cps at 1%)	- Ciprofloxacin hydrochloride - Dexamethasone	Children from 6 months	[111]
	OPHTHALMIC DROPS OR COLLYRIUMS	ALLERGY EYE DROPS- ketotifen fumarate solution/ drops	Benzalkonium chloride 0.01%, glycerine, purified water. may contain hydrochloric acid and/or sodium hydroxide (to adjust PH).	Ketotifen (0.025 %) (equivalent to ketotifen fumarate 0.035 %)	Children from 3 years. Children under 3 years of age: consult to doctor	[112]
	NASAL DROPS	LITTLE REMEDIES DECONGESTANT NASAL DROPS phenylephrine hydrochloride liquid	Benzalkonium chloride, glycerine, polyethylene glycol, potassium phosphate monobasic, purified water, Sodium EDTA, sodium phosphate dibasic	Phenylephrine hydrochloride 1.25 mg/mL	Children	[113]
	ORAL DROPS	BIO-G-TUSS PAEDIATRIC DROPS (solution)	Citric acid, grape flavour, glycerine, methylparaben, polyethylene glycol, propylparaben, purified water, Sodium citrate, sucralose	- Dextromethorphan HBr (7.5 mg/mL) - Guaifenesin (88 mg/mL) - Phenylephrine HCl (2.5 mg/mL)	Children	[114]
SOLI PREPARATIONS	CHEWABLE TABLET	Children’s Motrin—Ibuprofen Tablet, Chewable	Acesulfame potassium, ammonium glycyrrhizin, aspartame, carnauba wax, croscarmellose sodium, hypromellose, magnesium stearate, mannitol, natural and artificial flavours, silicon dioxide, sodium lauryl sulphate, soybean oil, succinic acid	Ibuprofen 100 mg	2 to 11 years	[115]
		Acetaminophen Children’s	Citric acid, crospovidone, D&C network No. 27 aluminium lake, D&C network No. 30 aluminium lake, dextrates hydrated, ethyl cellulose, flavours, magnesium stearate, mannitol, polyethylene, stearic acid, sucralose	Acetaminophen 80 mg	2 to 6 years	[116]
	TABLETS	Diazepam Tablet	Anhydrous lactose, magnesium stearate, cellulose microcrystalline, FD&C blue n. 1	Diazepam 10 mg	Children from 6 months	[117]
		Dexamethasone 1.5 mg tablet	Lactose monohydrate, magnesium stearate, maltodextrin, corn starch, sucrose	Dexamethasone 1.5 mg	It depends on the pathology	[118]

## Appendix F

**Table A5:** Liquid formulations for paediatric use in Research Articles.

Formula	Pharmaceutical Form	Excipients	Active Principle (Dose)	Age	Stability (Stability in Use)	References
Organic solvent-based formulation of lorazepam (Oral Solution)	Oral solution	PEG 400 (10% *v/v*), Propylene glycol (3% *m/v*), Glycerol (87% *v/v*) and Orange essence (0.1%)	Lorazepam (1 mg/mL)	Children 1 month to 12 years old	12 months at 4 °C (Stability in use: 4 weeks)	[119]
Oral solution of amlodipine besylate for children	Oral solution	Sucrose jarabe (32% *m/v*), Methylparaben (solution 15% *m/v*) (0.3% *m/v*) and Purified water (75%)	Amlodipine Besylate (0.5 mg/mL)	Paediatric Population (children and teenagers)	12 months at 4 °C (Stability in use: 18 weeks)	[120]
Oral tizanidine hydrochloride, Formulation for hospital use	Oral solution	CMC (carboxymethyl cellulose) (0.5%), Potassic sorbate (0.15%), Sucralose (0.10%), Citric acid and Purified water	Tizanidine Hydrochloride (1 g/mL)	Paediatric Population	70 days at 15–30 °C, 2–8 °C and 40 °C	[121]
Paediatric oral formulation of clonidine hydrochloride	Oral solution	Sucrose syrup (20% *v/v*), Raspberry essence (0.05%), Methyl paraben solution 15% (1% *m/v*), Citric acid monohydrate (1% *m/v*), Disodium hydrogen phosphate (1.8% *m/v*) and Purified water	Clonidine HCL (50 µg/mL)	Paediatric Population	9 months at room temperature, protected from light	[122]
Oral liquid formulation of clonidine hydrochloride for paediatric patients	Oral solution	Potassic sorbate, Sucrose and Monohydrate citric acid	Clonidine hydrochloride (20 µg/mL)	Paediatric Patients (all ages)	90 days at 5 °C (cooling) (Stability in use: 42 days at 5 °C)	[123]
Paediatric oral formulations of sodium dichloroacetate	Oral solution	Vehicle Mascagni (% *w/v*): Sucralose (0.02%), Hydroxyethyl cellulose (0.2%), Citric acid (0.09%), Sodium citrate (0.09%) and Potassium sorbate (0.18%)	Sodium dichloroacetate (DCA) (9.5% *w*/*v*)	Paediatric Patients	3 months at 4 °C and 25 °C (Stability in use: 1 month to 4 °C)	[124]
Furosemide solutions for personalized paediatric administration	Oral solution (extemporaneous)	Solution I: Buffer carbonate-bicarbonate (pH) (10 mL) Excipient for syrup (cps 100 mL) (ACOFARMA): sucrose, water, sorbitol, glycerine, aroma, citric acid, methyl paraben, potassium sorbate, sodium phosphate and colorant. Solution II: Buffer carbonate-bicarbonate (pH) (10 mL) -Excipient for syrup—without sugars (cps 100 mL) (ACOFARMA): sodium saccharine, xanthan gum, water, sorbitol, glycerine, aroma, citric acid, sodium citrate, methyl paraben, propyl paraben, potassium sorbate, sodium phosphate and colorant.	Furosemide (2 mg/mL)	Paediatrics	60 days at 4 and 25 °C	[125]
Formulation comprising acetaminophen, especially for paediatrics (PATENT)	Oral solution (nano-emulsion)	NF glyceryl mono linoleate (5–30%, preferably 8-26% *w/v*), PEG-35 castor oil (30–60%, preferably 39–46% *w/v*), NF diethylene glycol mono ethyl ether (20–45%, preferably 24–40% *w/v*) and Water	Paracetamol (5–18% *w*/*v*)	Paediatrics	NA	[126]
Paediatric formulations of ursodeoxycholic acid from oral administration	Oral suspension	Glycerol (20%), Methyl cellulose 1000 (1% *v/v*) and Purified water	Ursodeoxycholic acid (UDCA) (1.5 mg/mL)	Paediatric Population	30 days at 25 °C or in fridge	[127]
Oral paediatric formulation of hydrochlorothiazide	Oral suspension	Glycerol (20%), Methyl cellulose 1000 (1% *v/v*), Citric acid (pH corrector) and Water	Hydrochlorothiazide (2 mg/mL)	Paediatric Population in general	3 weeks at 5 °C and protected from light	[128]
Oral suspension of clindamycin HCL with ion exchange resin for paediatric use	Oral suspension	Glycerine (30% *w/v*), Sucralose (3%), Aroma of maple syrup (7%), Grape aroma (10%), Cremophor RH 40 (15%), Xanthan gum (0.2%) and Deionized water (cps 5 mL)	Clindamycin HCL resin (Amberlite IRP 69) (5.5% *w*/*v*)	Paediatric Population	1 month at 25 °C	[129,130]
Isoniazid suspension formulated with cationic resin for paediatric use	Oral suspension	Sorbitol solution 70% USP (4.9 mL/ 5 mL), USP monohydrate citric acid (50 mg/5 mL) and USP potassic sorbate (5 mg/5 mL)	Isoniazid resin/Kyron T-134 100 mg/5 mL/200 mg/5 mL	Paediatric Population	3 months at 40 °C (accelerated stability study)	[131]
Paediatric xylometazoline nasal spray formulation	Nasal Spray	Sodium colatum (105 mg/10 mL), PEG 400 (1.35 mL/10 mL), Sodium carboxy methyl cellulose (10 mg/10 mL), Glycerine (0.15 mL/10 mL), Methyl paraben (3.3 mg/10 mL), Sodium chloride and Purified water (cps 10 mL)	Xylometazoline HCl (5 mg/10 mL)	Paediatric Population	12 months at 25 °C	[132]
